# Kynurenic Acid Induces Impairment of Oligodendrocyte Viability: On the Role of Glutamatergic Mechanisms

**DOI:** 10.1007/s11064-016-2009-7

**Published:** 2016-07-21

**Authors:** Ewa Langner, Marta K. Lemieszek, Jacek M. Kwiecień, Grażyna Rajtar, Wojciech Rzeski, Waldemar A. Turski

**Affiliations:** 1 Department of Medical Biology, Institute of Rural Health, Lublin, Poland; 2 Department of Pharmacology, Medical University, Lublin, Poland; 3 Department of Pathology and Molecular Medicine, M. deGroote School of Medicine, McMaster University, Hamilton, Canada; 4 Department of Clinical Pathomorphology, Medical University, Lublin, Poland; 5 Department of Virology and Immunology, Institute of Microbiology and Biotechnology, Maria Curie-Sklodowska University, Lublin, Poland; 6 Department of Experimental and Clinical Pharmacology, Medical University, Lublin, Poland

**Keywords:** Oligodendrocytes, OLN-93, Kynurenic acid (KYNA), Myelin, Cell viability, Glutamate receptors

## Abstract

Kynurenic acid (KYNA) is an end stage product of tryptophan metabolism with a variety of functions in the human body, both in the central nervous system (CNS) and in other organs. Although its activity in the human brain has been widely studied and effects on neural cells were emphasized, the effect of KYNA on oligodendroglial cells remains unknown. Present study aims at describing the activity of high concentration of KYNA in OLN-93 cells. The inhibition of OLN-93 oligodendrocytes viability by KYNA in a medium with reduced serum concentration has been demonstrated. Although decreased metabolic activity of KYNA treated OLN-93 cells was shown, the cells proliferation was not altered. KYNA treatment did not alter morphology as well as expression level of cell cycle and proliferation regulating proteins. Furthermore, glutamate receptor antagonists and agonists did not alter the inhibitory effect of KYNA on viability of OLN-93 oligodendrocytes. This study contributes to the elucidation of effects of KYNA on oligodendrocytes in vitro, yet further analyses are necessary to explain the mechanisms behind the damage and loss of myelin sheaths.

## Introduction

Kynurenine pathway (KP) serves as the main route of tryptophan metabolism resulting in nicotinamide adenine dinucleotide (NAD+) as well as so-called kynurenines formation. Thus far, it has been well established that kynurenines exert key biological functions in the mammalian central nervous system (CNS) being able to affect the activity of several glutamate receptors. Remarkable changes in the ratio between tryptophan and its metabolites are often the hallmark of a variety neurological disorders i.e. Huntington’s disease, Alzheimer’s disease, Parkinson’s disease, seizure disorders, dementia, schizophrenia, and depression [1, 2].

Kynurenic acid (KYNA) is one of the catabolic end stage products of KP and its’ neuroprotective role in the brain is well recognized. This endogenous tryptophan metabolite is usually found in the brain at nanomolar concentrations and is known to exert anticonvulsant and sedative activities [1, 3].

Although the effects of KYNA on neural cells, both under physiological and pathological conditions have been excessively studied [2], its’ effect on oligodendroglial cells and on the myelin sheath is poorly understood. The recent study by Dabrowski and colleagues shows myelin damage and loss in the spinal cord of rats continuously infused with high doses of KYNA [4]. Importantly, the loss of myelin was not associated with inflammatory infiltration, death of oligodendrocytes or damage to naked axons indicating a specific but yet unknown effect of KYNA on oligodendrocytes. The present study aims to evaluate the influence of KYNA on OLN-93 oligodendroglial cells in vitro and to establish the role of glutamatergic mechanism in this process.

## Materials and Methods

### Drugs and Reagents

If not indicated otherwise, chemicals used in the study were purchased from Sigma–Aldrich Co. (St. Louis, MO, USA). CPPene ((*R*)-4-[(*E*)-3-phosphonoprop-2-enyl]piperazine-2-carboxylic acid) was obtained from Sandoz (Basel, Switzerland). AMPA ((S)-α-Amino-3-hydroxy-5-methyl-4-isoxazolepropionic acid) and ATPA ((RS)-2-Amino-3-(3-hydroxy-5-tert-butylisoxazol-4-yl)propanoic acid) were purchased from Tocris Bioscience (Bristol, UK). All chemicals except KYNA, AMPA, ATPA and GYKI 52466 were dissolved in phosphate buffered saline (PBS) to prepare stock solutions as follow: glutamate 0.5 M, NMDA 50 mM, CPPene 25 mM, MK-801 10 mM. KYNA 0.5 M was dissolved in 1 N NaOH followed by PBS addition (final concentration of NaOH in stock solution and culture medium did not exceed 68 and 0.136 %, respectively). AMPA was dissolved in 1 N HCl followed by PBS addition (final concentration of HCl in stock solution and culture medium did not exceed 20 and 0.04 %, respectively). GYKI 52466 was dissolved in 5N HCl followed by PBS addition (final concentration of HCl in stock solution and culture medium did not exceed 2 and 0.02 %, respectively). ATPA 150 mM was dissolved in DMSO followed by addition of PBS (final concentration of DMSO in stock solution and culture medium did not exceed 50 and 0.067 %, respectively). Working solutions were prepared each time in fresh culture medium. In addition, the maximal concentration of the solvent was tested in cultures and no changes in cell growth and morphology were observed.

### Cell Culture

Oligodendrocyte cell line OLN-93 was obtained from Department of Neonatolgy, Charité-Virchow Clinics, Humboldt University, Berlin, Germany and maintained in DMEM/F12 HAM (1:1) **(**Dulbecco’s Modified Eagle’s Medium/Nutrient mixture F-12 HAM) in a humidified atmosphere of 95 % air and 5 % CO2 at 37 °C. Culture medium was supplemented with 10 % fetal bovine serum (FBS), penicillin (100 U/ml) and streptomycin (100 μg/ml).

### Cell Viability Assessment––MTT Assay

Cells viability was determined with use of MTT (3-(4,5-dimethylthiazol-2-yl)-2,5-diphenyltetrazolium bromide) assay. Cells were seeded onto 96-well microplates at a density of 1 × 10^4^ cells/well. The next day, the medium was replaced with the fresh one with reduced FBS to a concentration of 2 % (control) or with indicated concentrations of KYNA, glutamate antagonists (CPPene, MK-801 and GYKI 52466) and glutamate agonists (glutamate, NMDA, AMPA and ATPA). After 24 h of incubation, MTT solution (5 mg/ml) was added (final concentration of 0.75 mg/ml) and incubated for 3 h. Resultant formazan crystals were solubilized overnight in sodium dodecyl sulfate (SDS, Sigma Aldrich) buffer (SDS in 0.01 N HCl). Absorbance was recorded on a microplate reader (BioTek ELx800, Winooski, VT, USA) at the wavelength of 570 nm.

### Cell Proliferation Assessment––MTT Assay

Cells were seeded onto 96-well microplates at a density of 2.5 × 10^3^ cells/well. The next day, the medium was replaced with the fresh one alone (control) or with indicated concentrations of KYNA (10, 50, 100, 250, 500, 1000 µM). After 48 or 96 h of incubation, MTT solution (5 mg/ml) was added (final concentration of 0.75 mg/ml) and incubated for 3 h. Resultant formazan crystals were solubilized overnight in sodium dodecyl sulfate (SDS, Sigma Aldrich) buffer (SDS in 0.01 N HCl). Absorbance was recorded on a microplate reader (BioTek ELx800, Winooski, VT, USA) at the wavelength of 570 nm.

### Cell Proliferation Assessment––BrdU Assay

Cells were seeded onto 96-well microplates at a density of 2.5 × 10^3^ cells/well. The next day, the medium was replaced with the fresh one alone (control) or with indicated concentrations of KYNA (1, 5, 10, 25, 50, 100, 250, 500, 1000 µM). After 48 h of incubation, BrdU was added and the following steps were performed according to the manufacturer’s procedures (Cell Proliferation ELISA BrdU, Roche Diagnostics GmbH, Penzberg, Germany). Absorbance was measured at a wavelength of 450 nm using microplate reader (BioTek ELx800).

### Cell Morphology/Cytoskeleton Visualization

The morphology of oligodendroglial cells treated with KYNA was shown by F-actin filament staining with use of rhodamine-conjugated phalloidin (RHPH). Cells grown on chamber slides (Nunc) coated with poly-d-lysine (50 µg/ml) were subjected to 500 μM KYNA (in a medium with reduced concentration of FBS to 2 %) for 24 h. Then, the cells were rinsed twice with PBS and fixed with 3.7 % paraformaldehyde for 20 min, followed by 5 min permeabilization in 0.1 % Triton X−100. Next, half-hour incubation with phalloidin-rhodamine (66 pmol/ml) was performed. Nuclei were counterstained for 5 min with Hoechst 33342 (0.24 μg/mL). Cell images were captured with fluorescence microscopy (Olympus BX51 System Microscope; Olympus Optical Co., Ltd., Tokyo, Japan, and CellFamily AnalySIS software) at ×400 magnification.

### Assessment of Cell Death–Cell Death Detection ELISA

Cells were seeded onto 96-well microplates at a density of 1 × 10^4^ cells/well. The next day, the medium was replaced with the fresh one alone (control) or with 500 μM KYNA for 24 h. Next, the following steps were performed according to the manufacturer’s procedures of Cell Death Detection ELISAPLUS kit (Roche Diagnostics). Absorbance was measured at 405 nm wavelength using microplate reader (BioTek ELx800).

### Immunobloting

For western blot analysis 500 µM KYNA (in a medium with reduced to a concentration of 2 % FBS) was used. OLN-93 cells after treatment were washed with ice-cold PBS, harvested with 5 mM EDTA and lysed for 1 h in ice-cold lysis buffer consisting of 1 % NP40, 0.5 % sodium deoxycholate, 0.1 % SDS, 1 mM EDTA, 1 mM EGTA, 1 mM Na_3_VO_4_, 20 mM NaF, 0.5 mM DTT, 1 mM PMSF and protease inhibitor cocktail in PBS pH 7.4. After centrifugation in 4 °C (14,000×*g* for 10 min) supernatants were collected and solubilized in 6× Laemmli sample buffer (0.5 M Tris/HCl pH 6.8, 30 % glycerol, 10 % SDS, 5 % β-merkaptoetanol, 0.012 % bromophenol blue). Forty micrograms of total protein were separated by SDS–PAGE (10–14 % SDS–polyacrylamide gel) and then transferred into PVDF membrane (Millipore, Billerica, MA, USA). The blots were probed overnight with indicated primary antibodies phospho-ERK1/2 (Thr^202^/Tyr^204^, 1:1000), phospho-Akt (Ser^473^, 1:1000), cyclin D1 (1:2000), CDK4 (1:1000), CDK6 (1:1000) and β-actin (1:1000) from Cell Signaling Technology, Beverly, MA, USA and p21 (1:1000) from Santa Cruz Biotechnology, Dallas, TX, USA. Primary antibodies were then detected with HRP-conjugated secondary antibodies (1:2000; Cell Signaling Technology). The visualization of the proteins was performed using an enhanced chemiluminescence detection system (Pierce, Rockford, IL, USA).

### Statistical Analysis

The data were plotted as the mean ± SD with use GraphPad Prism 5 (GraphPad Software, Inc., La Jolla, CA, USA).

## Results

### Effect of KYNA on Viability of OLN-93 Cells In Vitro

**Fig. 1:**
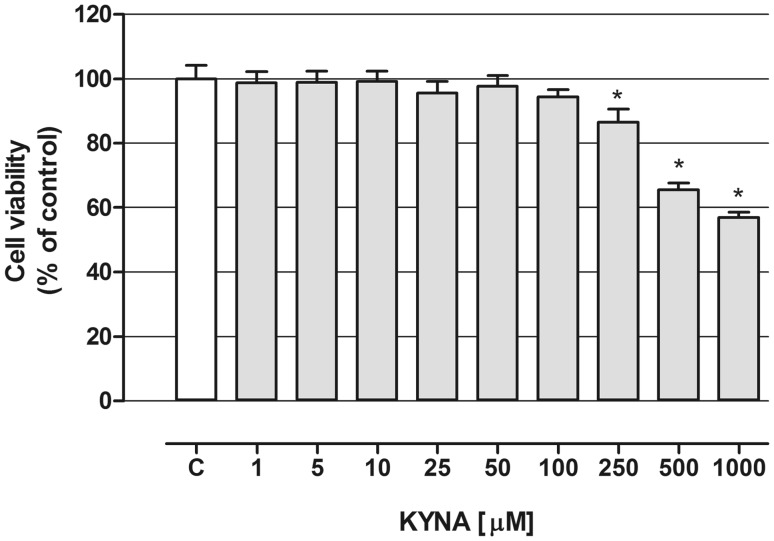
Effect of KYNA on viability of OLN-93 cells in vitro. OLN-93 cells were exposed to indicated concentrations of KYNA (in a medium with reduced to 2 % FBS) and viability was measured after 24 h by MTT test. The values were means ± SD; n = 6. *At least p < 0.05 vs. control (one-way ANOVA test, post test: Tukey), *C* control

In the first set of experiments evaluation of OLN-93 cells viability was performed upon KYNA treatment. Cells were exposed to sequential dilutions of KYNA for 24 h in a medium containing 2 % FBS and MTT test was performed. Decreased viability of cells was observed upon treatment with KYNA in a concentration range: 250–1000 µM (Fig. 1). The highest concentration tested decreased cell viability over 40 % vs control conditions.

### Effect of KYNA on Proliferation and Morphology of OLN-93 Cells In Vitro

**Fig. 2:**
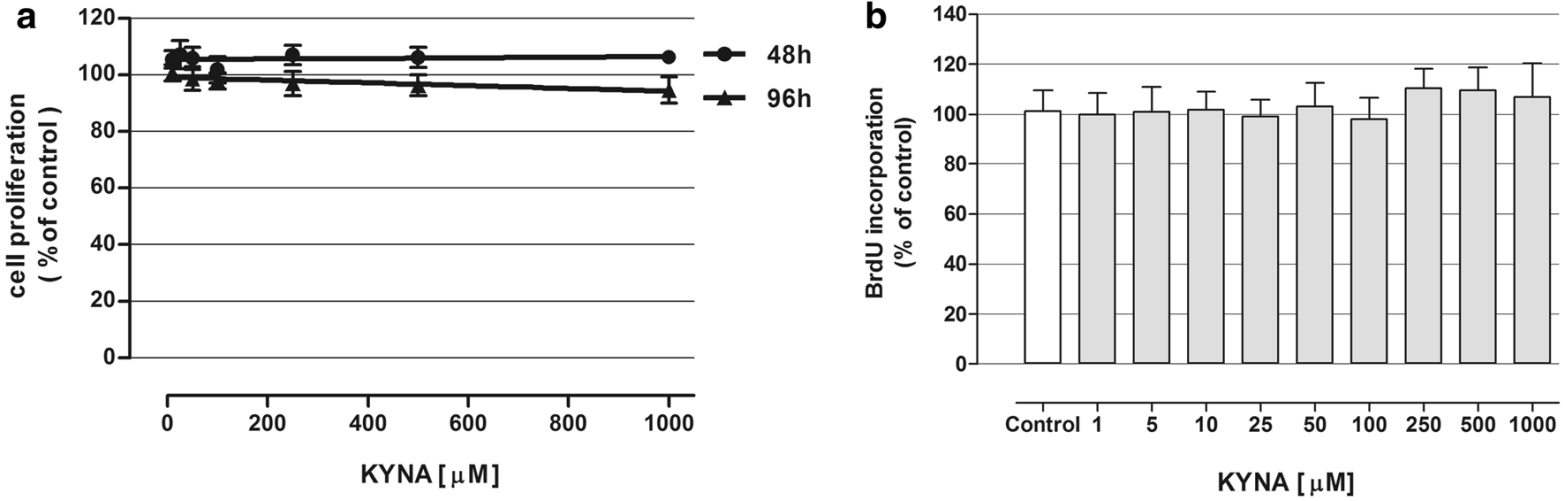
Effect of KYNA on proliferation of OLN-93 cells in vitro. **a** OLN-93 cells were exposed to indicated concentrations of KYNA (in a medium with 10 % FBS) for 48 or 96 h and proliferation was measured by MTT test. The data were means ± SD; n = 6 and were analyzed by means of linear regression. **b** OLN-93 cells were exposed to indicated concentrations of KYNA (in a medium with 10 % FBS) for 48 h and proliferation was assessed by BrdU test. The values were means ± SD; n = 18. *At least p < 0.05 vs. control (one-way ANOVA test, post test: Tukey), *C* control

In order to assess the impact of KYNA on oligodendroglial cells’ growth/proliferation, MTT test was used upon 48 and 96 h of incubation with tested compound in a medium containing 10 % FBS. No statistically significant effect on OLN-93 cells proliferation has been shown (Fig. 2a). Similarly, BrdU incorporation-based test has shown no alteration in DNA synthesis within OLN-93 cells in vitro after 48 h incubation with 1–1000 µM concentrations of KYNA (Fig. 2b).

**Fig. 3:**
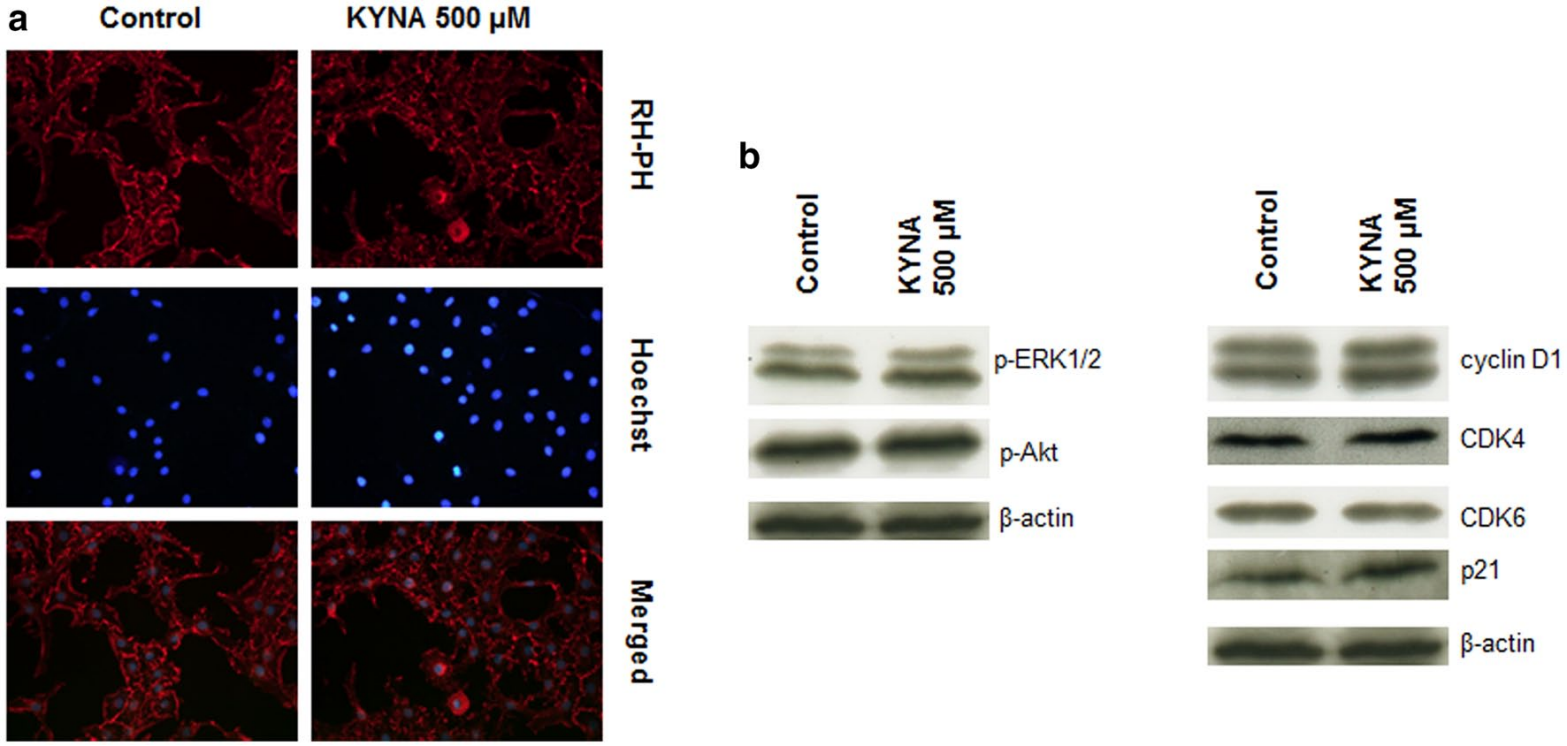
Effect of KYNA on OLN-93 cell morphology and expression of proteins regulating proliferation and cell cycle. **a** OLN-93 cells after 24 h exposure to medium alone (control) or KYNA 500 µM were fixed and stained with rhodamine-conjugated phalloidin (*red*); nuclei were counterstained with Hoechst 33342 (*blue*). Images were captured at ×400 magnification. **b** OLN-93 cells were incubated for 24 h with medium alone (Control) or KYNA 500 µM, harvested and lysed. 40 µg of total protein was separated by SDS–PAGE, transferred on PVDF membranes and expression of p-ERK1/2 (Thr^202^/Tyr^204^), p-Akt (Ser^473^), cyclin D1, CDK4, CDK6 and p21 proteins was visualized by western blot analyses. β-actin was used as an internal control

To further explore the influence of KYNA on the cell morphology, actin filaments were visualized with RHPH. No changes in cytoskeleton composition and morphology of OLN-93 cells upon KYNA 500 µM treatment were noted (Fig. 3a). Moreover, no cell death induction, neither apoptosis nor necrosis, was observed due to KYNA treatment (data not shown).

In order to evaluate whether KYNA affects expression of proteins regulating cells’ proliferation and cell cycle, western blot analyses were performed. No significant changes in phosphorylation level of extracellular-signal regulated kinases 1/2 (ERK1/2) on Thr^202^/Tyr^204^ and protein kinase B (Akt) on Ser^473^ were noted (Fig. 3b left panel). No meaningful changes in expression of both cell cycle stimulators i.e. cyclin D1, cyclin-dependent kinases CDK4, CDK6 and cell cycle inhibitor p21 protein were detected upon KYNA 500 µM incubation (Fig. 3b right panel).

### Effect of Glutamate Receptor Antagonists on Activity of KYNA on OLN-93 Cells In Vitro

**Fig. 4:**
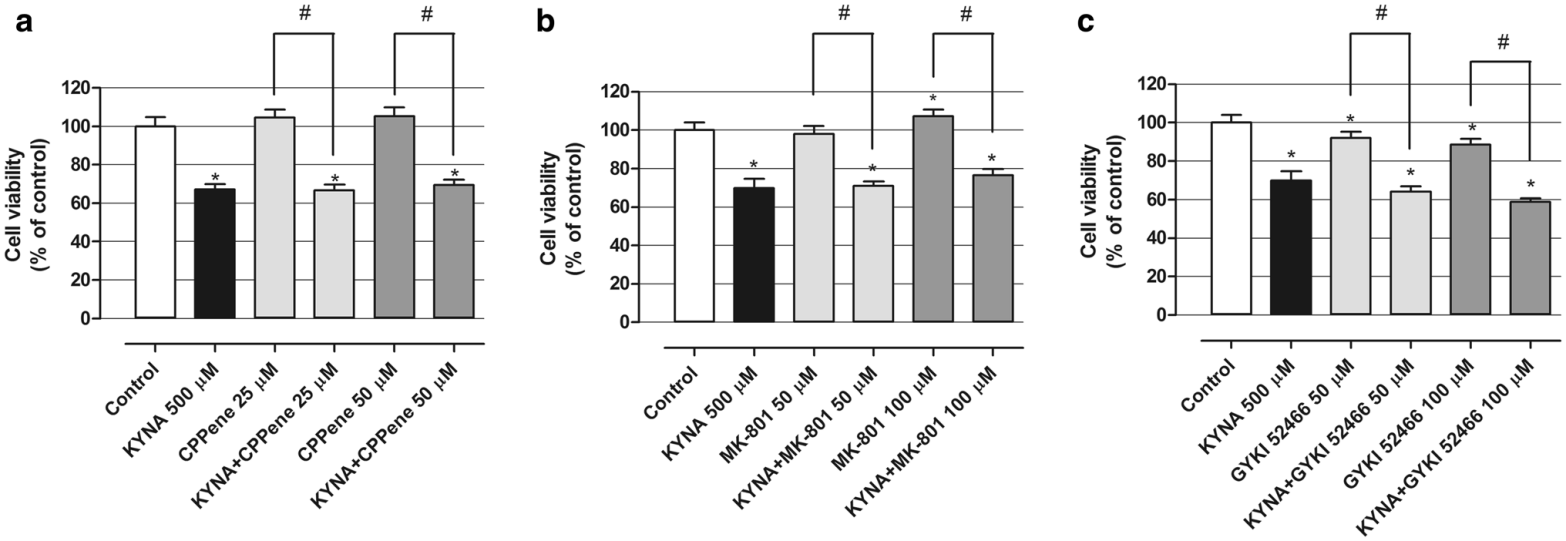
Effect of glutamate receptor antagonists on KYNA activity in OLN-93 cells in vitro. OLN-93 cells were incubated for 24 h with indicated concentrations of KYNA, CPP, MK-801 and GYKI 52466 alone or in combination: KYNA 500 µM + glutamate receptor antagonist (in a medium with reduced to 2 % FBS) and viability was measured by MTT test. The values were means ± SD; n = 6. *At least p < 0.05 vs. Control; ^#^at least p < 0.05 (one-way ANOVA test, post test: Tukey)

Glutamate receptor antagonists including CPPene (25, 50 µM), MK-801 (50, 100 µM) and GYKI 52466 (50, 100 µM) were used alone or in combination with KYNA (500 µM) and viability of treated oligodendroglial cells was assessed after 24 h with MTT test. All of tested antagonists did not stop the effect of KYNA that decreased viability of OLN-93 cells (Fig. 4a–c). Moreover, CPPene did not induce changes in cells’ viability when applied separately (Fig. 4a). Viability of cells treated with MK-801 and GYKI 52466 was slightly changed, however, the influence of applied antagonists was opposite. MK-801 in concentration of 100 µM increased cell viability by 7.4 % (Fig. 4b). GYKI 52466 at 50 and 100 µM decreased cell viability by 7.9 and 11.4 %, respectively (Fig. 4c).

### Effect of Glutamate Receptor Agonists on KYNA Activity in OLN-93 Cells In Vitro

**Fig. 5:**
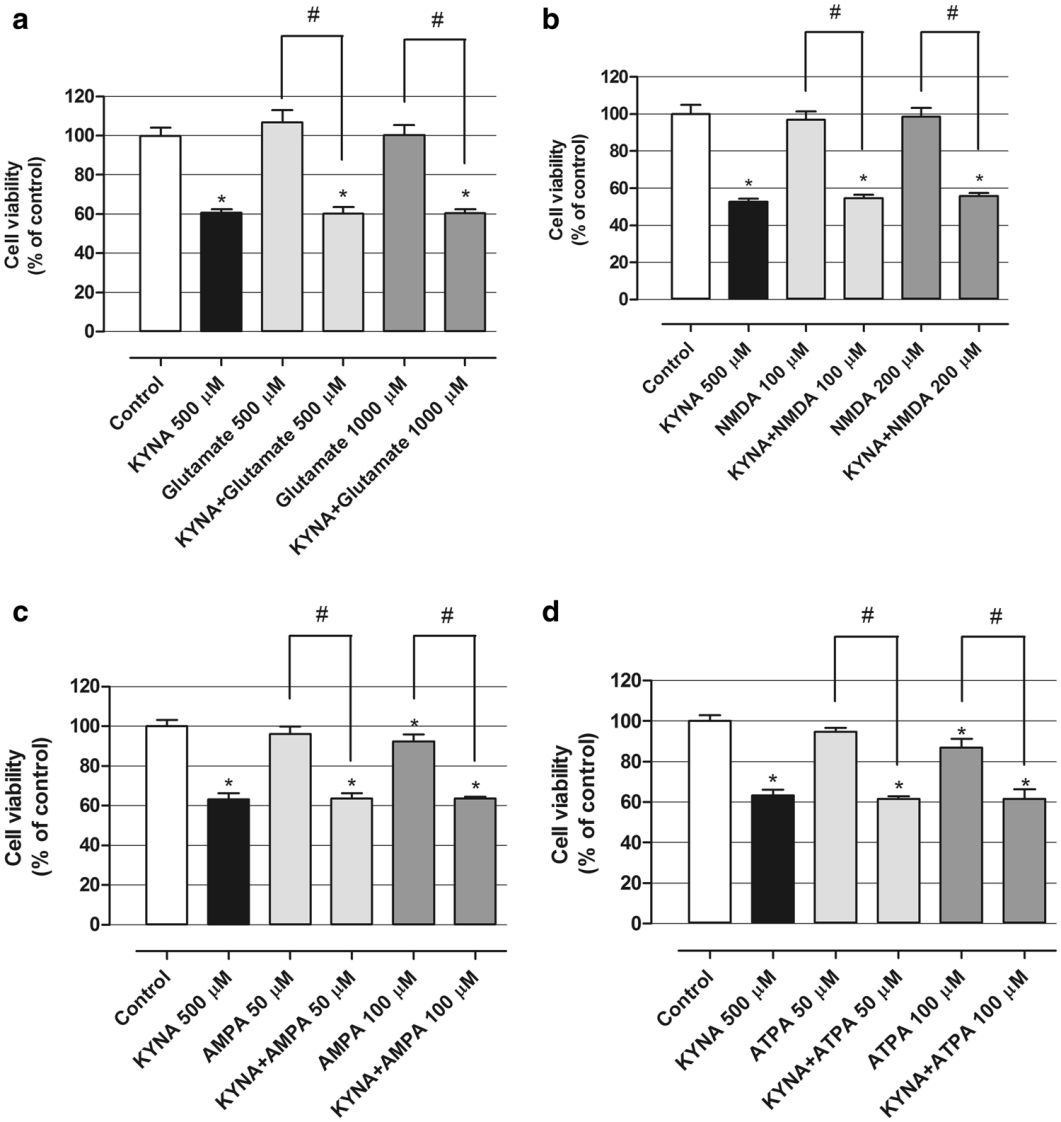
Effect of glutamate receptor agonists on KYNA activity in OLN-93 cells in vitro. OLN-93 cells were incubated for 24 h with indicated concentrations of KYNA, glutamate, NMDA, AMPA and ATPA alone or in combination: KYNA 500 µM + glutamate receptor agonist (in a medium with reduced to 2 % FBS) and viability was measured by MTT test. The values were means ± SD; n = 6. *At least p < 0.05 vs. Control; ^#^at least p < 0.05 (one-way ANOVA test, post test: Tukey)

Glutamate receptor agonists including glutamate (500, 1000 µM), NMDA (100, 200 µM) and AMPA (50, 100 µM) were used alone or in combination with KYNA (500 µM) for 24 h and the viability of treated oligodendroglial cells was assessed with the MTT test. All tested agonists applied alone did not affect the viability of OLN-93 cells in vitro (Fig. 5a, b) with except for AMPA at 100 µM and ATPA at 100 µM, where viability of oligodendroglial cells was reduced by 7.6 and 13.2 %, respectively (Fig. 5c, d). Furthermore, none of the used glutamate receptor agonists influenced the inhibitory action of KYNA on OLN-93 cells.

## Discussion

The route and function of KP and its metabolites are well established in neurons but not in oligodendrocytes. Despite the demonstrated lack of expression of indoleamine 2,3-dioxygenase (IDO-1) and tryptophan 2,3-dioxygenase (TDO-2) in primary human oligodendrocytes [5], the details of kynurenines activity within oligodendroglial cells is still being explored. Recently, Dabrowski and colleagues demonstrated morphologically damage and loss of myelin sheets after a 5–7 days subdural infusion of high concentrations of KYNA with no apparent death of oligodendrocytes [4]. Therefore, in the present study we have performed a series of experiments aiming to elucidate the mechanism of KYNA activity on oligodendrocytes in vitro.

A permanent OLN-93 cell line obtained from spontaneously transformed cells in primary cultures of rat brain [6] was used in the study. The cells were shown to resemble the features of primary oligodendrocytes with the ability to differentiate into late immature oligodendrocytes, depending on serum concentration in the growth medium [6, 7]. Furthermore, previous study of our group demonstrated kynurenine aminotransferases I and II (KAT I and KAT II) expression in OLN-93 cells as well as the ability of these cells to synthesize KYNA from exogenously added l-kynurenine [8]. Here, we have explored the influence of exogenously added KYNA on OLN-93 cells viability and proliferation. A reduction in cell viability has been noted in cultures incubated with increasing concentrations of KYNA. Concomitantly, no changes in cell proliferation have been indicated which had been screened both by metabolic activity and BrdU incorporation assessment. Discrepancy in the observed metabolic activity of cells when tested for cell viability and cell proliferation may be linked to different serum concentration in the incubation medium, reduced (2 %) or standard (10 %), respectively. According to several studies describing intracellular modifications in OLN-93 cells in low-serum and serum-deprivation conditions [6, 7, 9] we cannot exclude the influence of various culture conditions on our study results. However, taking into consideration described measurements as well as microscopic observations of KYNA treated OLN-93 cells, we may clearly conclude that KYNA (1–1000 µM) does not affect OLN-93 cells proliferation, DNA synthesis and morphology, yet in concentration range 250–1000 µM it downregulates viability/metabolic activity of oligodendrocytes grown in a medium with a reduced serum concentration. This is consistent with the results of Dabrowski et al. [4] demonstrating damage and loss of myelin sheets in the spinal cord of rats after intrathecal KYNA infusion with a simultaneous lack of oligodendrocytes injury [4]. Apparently KYNA reduces the ability of oligodendrocytes to maintain the myelin sheaths due to the reduction in a metabolic activity that still needs to be characterized. Present results are unlike our earlier observations concerning antiproliferative activity of KYNA in a broad spectrum of human cancer cell lines i.e. T98G glioma (IC_50_ value of 1.3 mM), HT-29 colon adenocarcinoma (IC_50_ value of 0.9 mM) and Caki-2 renal carcinoma (IC_50_ value of 0.04 mM) [10–12] as well as normal rabbit synoviocytes HIG-82 in vitro (IC_50_ value of 5.9 mM) [13].

In the attempt to elucidate molecular mechanisms of KYNA-related reduction of cell viability, the expression of several proteins engaged in the regulation of cellular proliferation and cell cycle was explored. ERK1/2 and Akt kinases are key regulatory proteins of basic cell functions i.e. proliferation, growth and differentiation or apoptosis. Phosphorylation of Thr^202^/Tyr^204^ and Ser^473^ sites is necessary for the activation of ERK1/2 and Akt kinases, respectively [14, 15]. It can be concluded that KYNA did not act through the ERK1/2 and Akt regulated signaling pathways, since incubation of cells with KYNA did not significantly influence the phosphorylation level of ERK1/2 and Akt kinases. Furthermore, no meaningful changes in expression of cell cycle regulatory proteins [16] were observed i.e. cyclin D1, CDK4, CDK6 as well as cell cycle inhibitor, p21 protein. This may be consistent with the observations of no influence of KYNA on OLN-93 cells proliferation and growth shown in this study.

Recently, Benjamins and colleagues demonstrated the effect of KYNA on reduction in oligodendrocyte viability in primary cultures from the neonatal rat brain [17]. Also Lisak et al. observed the same effect in oligodendrocyte precursor cells (OPCs) derived from neonatal rat brain [18]. However, in both studies the effect of only a single concentration of 25 µM KYNA was investigated [17–19] while we evaluated concentration-dependent activity of KYNA in a broad range of concentrations from 1 to 1000 µM. Furthermore, Benjamins et al. [17] and Lisak et al. [18] demonstrated that KYNA treatment leads to cell death of OLs and OPCs of primary cultures which has been shown with trypan blue staining [17–19]. Although we did not applied the trypan blue exclusion, the results of our study suggest a decrease in metabolic activity of OLN-93 cells due to KYNA treatment with no hallmarks of cell toxicity and death. The discrepancy in findings may be explained by the usage of differential cell cultures, primary vs. immortalized, in which cells may vary in differentiation stage. Morphological observations in vivo support the notion of the reduction of metabolic activity of oligodendrocytes that appeared shrunken, poor in organelles and with reduced perikaryon and processes, cell death however, was not prominent [4].

Since KYNA is a broad spectrum antagonist of ionotropic glutamate receptors [20, 21], to further explore mechanism of its action exerted on oligodendrocytes the effect of drugs affecting glutamate receptors was investigated. CPPene, a selective antagonist of the *N*-methyl-d-aspartate (NMDA) receptor did not affect viability of OLN-93 cells. However, MK-801, an uncompetitive antagonist of NMDA receptor slightly enhanced viability of OLN-93 cells at the higher tested concentration. In contrast, GYKI 52466, a non-competitive antagonist of α-amino-3-hydroxy-5-methyl-4-isoxazolepropionic acid (AMPA) receptor marginally reduced metabolic activity of oligodendrocytes in both investigated concentrations. Importantly, all three studied glutamate antagonists did not modify cell viability decreasing effect of KYNA. In next series of experiments glutamate agonists were employed. Glutamate, an endogenous non-selective agonist and NMDA, a selective agonist of NMDA receptor affected neither viability of OLN-93 cells when applied alone nor modified the action of KYNA. Unexpectedly, agonists of AMPA receptor AMPA and ATPA at the higher studied concentration slightly decreased viability of OLN-93 measured by means of MTT method. However, neither AMPA nor ATPA affected effects exerted by KYNA. Taking together, since glutamate receptor antagonists did not reduce viability of OLN-93 in vitro, as KYNA did, and glutamate agonists did not antagonize the effect of KYNA it seems reasonable to state that KYNA reduces viability of oligodendrocytes through the mechanism distinct from its interaction with glutamate receptors. According to results obtained by Gerstner and colleagues, OLN-93 cells lack the expression of several subunits of NMDA receptor i.e. NR1, NR2A and NR2B, which may suggest no functional activity of NMDA receptors [22] and may explain no or marginal effect of several glutamate receptor antagonists and agonists on oligodendrocytes in our study. However, previous study characterizing KYNA synthesis in OLN-93 cells showed that KYNA production had been altered due to glutamate and AMPA treatment, suggesting their interaction with adequate receptors. Similarly to our observations, no influence of NMDA had been shown [8]. Furthermore, a research performed on OLN-93 cells indicated protective effect of NMDA antagonist MK-801 and AMPA antagonist 2,3-dioxo-6-nitro-1,2,3,4-tetrahydrobenzo[f]quinoxaline-7-sulfonamide (NBQX) on OLN-93 cells during oxygen-glucose deprivation/reperfusion [23] implying functional activity of both NMDA and AMPA receptors.

Apart from action on glutamate receptors KYNA is known as α7 nicotinic acetylcholine (α7nACh) receptor antagonist [24] and an agonist of G-protein receptor GPR35 [25]. Its action on aryl hydrocarbon receptor (AhR) is disputable due to conflicting reports [26, 27]. Moreover, it has been reported that KYNA reduced respiratory parameters in isolated rat heart mitochondria [28]. Further studies are needed to fully elucidate the mechanism by which KYNA affects metabolic activity of oligodendrocytes.

In summary, in an in vitro study we showed that KYNA at high levels reduces the viability of oligodendrocytes by mechanisms distinct from that of mediated by glutamatergic receptors. Our results support the recent observation that prolonged subdural administration of high concentrations of KYNA in rats produced damage and loss of myelin sheaths with no concurrent necrosis of oligodendrocytes.
